# Integrating Visualizations into Modeling NEST Simulations

**DOI:** 10.3389/fninf.2015.00029

**Published:** 2015-12-17

**Authors:** Christian Nowke, Daniel Zielasko, Benjamin Weyers, Alexander Peyser, Bernd Hentschel, Torsten W. Kuhlen

**Affiliations:** ^1^Visual Computing Institute, RWTH Aachen University, Jülich Aachen Research Alliance - High-Performance ComputingAachen, Germany; ^2^Simulation Lab Neuroscience - Bernstein Facility for Simulation and Database Technology, Institute for Advanced Simulation, Jülich Aachen Research Alliance, Forschungszentrum Jülich GmbHJülich, Germany

**Keywords:** interactive visualization, spiking neural network modeling, workflow integration, data management, coordinated and multiple views

## Abstract

Modeling large-scale spiking neural networks showing realistic biological behavior in their dynamics is a complex and tedious task. Since these networks consist of millions of interconnected neurons, their simulation produces an immense amount of data. In recent years it has become possible to simulate even larger networks. However, solutions to assist researchers in understanding the simulation's complex emergent behavior by means of visualization are still lacking. While developing tools to partially fill this gap, we encountered the challenge to integrate these tools easily into the neuroscientists' daily workflow. To understand what makes this so challenging, we looked into the workflows of our collaborators and analyzed how they use the visualizations to solve their daily problems. We identified two major issues: first, the analysis process can rapidly change focus which requires to switch the visualization tool that assists in the current problem domain. Second, because of the heterogeneous data that results from simulations, researchers want to relate data to investigate these effectively. Since a monolithic application model, processing and visualizing all data modalities and reflecting all combinations of possible workflows in a holistic way, is most likely impossible to develop and to maintain, a software architecture that offers specialized visualization tools that run simultaneously and can be linked together to reflect the current workflow, is a more feasible approach. To this end, we have developed a software architecture that allows neuroscientists to integrate visualization tools more closely into the modeling tasks. In addition, it forms the basis for semantic linking of different visualizations to reflect the current workflow. In this paper, we present this architecture and substantiate the usefulness of our approach by common use cases we encountered in our collaborative work.

## 1. Introduction

In recent years, advances in simulation technology and computing power have made simulation of large-scale spiking neural networks feasible. These simulations produce an immense amount of data that needs to be analyzed by researchers in order to validate the simulated models. In order to assist the analysis process of simulation output, computational neuroscience resorts to interactive visualization methods to leverage humans' abilities for pattern recognition, intuition, and creativity. However, neural simulations produce a multitude of data modalities, e.g., spike trains, connectivity data, and derived metrics, on multiple scales, to only name a few. Therefore, a successful visualization will have to provide integration of these modalities into a unifying solution embedded into the workflow of modeling neural simulations.

One way to address this challenge is the use of the coordinated multiple views (CMVs) paradigm (cf. North and Shneiderman, 1997; Wang Baldonado et al., 2000). CMV systems have successfully been used to uncover complex relationships in data by enabling users to relate different data modalities and scales (cf. Ryu et al., 2003), thereby assisting researchers in context switches, comparative tasks, and supplementary analysis techniques. To relate these different data modalities, coordination between views, especially *linking*, is required. Linking refers to the idea of connecting different views in such a way that, if the user interacts with one view, this will affect all other views in the same semantic way—e.g., selecting an entity in one view will also highlight all occurrences of the same entity in all other views. However, this approach requires, on the one hand, coherent access to data so that all views display the same data model, and, on the other, a synchronization mechanism for shared data entities across views (i.e., selections).

In order to develop a software architecture that applies the CMV paradigm on data resulting from neural simulations, we first observe the researchers' workflow of modeling simulations, the resulting data modalities, and relationships between these artifacts. Subsequently, we derive requirements for a system that enables coupling of multiple visualizations. Collecting these requirements necessitates an interdisciplinary approach between computational neuroscientists and visualization experts. To this end, meetings were conducted in which we presented the progress of our development and collected feedback, new requirements, or new visualization designs for new hypothesis about the data. A key observation from this process is a highly volatile analysis workflow of modeling neural systems resulting from ever changing hypotheses about the simulated data. Hence, an architecture must provide access and processing of data to meet this flexibility.

The main contribution of this paper is a software infrastructure that provides a concept to access simulation data for further processing and visualization purposes which has been driven by a close cooperation between neuroscientists and visualization experts. It provides synchronization capabilities between various visualizations which can be used in dynamic workflows and can be embedded into the work environment of the scientists in order to access, modify, and process stored data artifacts conveniently. Moreover, it forms the basis for an architecture enabling semantical linking of multiple visualizations, based on the current workflows and intents of its users, in the future. We demonstrate the applicability of this approach by presenting use cases which deploy the proposed system.

In the following paragraphs, we will first introduce related work focusing on CMVs. Following this, we present existing integrations of visualizations for data analysis.

CMVs is a visualization technique intended to support exploratory data analysis (cf. Roberts, 2007). The overall idea is to offer interaction with different representations of the same data, while emphasize different details to understand the data. The challenge within utilizing this approach is the coordination of views, because coordination depends on the inter-related information underlying the visual analysis task which in turn is domain-specific. In addition, users often need unforeseen combinations of coordination that depend heavily on the data to explore. To this end, Weaver (2004) introduces *Improvise*, a system which allows the user to build multiple coordinated views interactively by means of shared-object coordination. In addition, Improvise provides an expression-based visual abstraction language that enables users to describe the relationships of their data to allow a fine-grained control of coordination mechanisms between them. However, Improvise is restricted to the visual representation of relational data, which does not cover all heterogeneous data produced in modeling neural systems. North and Shneiderman (2000) approach the problem in their tool *Snaptogether* by providing a user interface where a formal description of related data items is specified by the user. This makes it possible to enrich visualizations by additional views without programming abilities. Moreover, they provided an API for extending the framework with additional views. However, similar to Improvise, the introduced framework is built around relational data. Boukhelifa and Rodgers (2003) describe a model and software system called *CViews* for multiple views which formalizes coordination concepts. The model is designed to be generic without any bias toward navigation concepts, requirements on data modalities, and communication paradigms used to synchronize data between views. However, our work, while borrowing some of these ideas, focuses foremost on establishing a synchronization paradigm for simulation data and visualization entities rather addressing a complete abstract approach as presented by the authors. Nevertheless, it forms a basis for future concepts driven by a description of the coordination's semantic.

Sousa and Aguiar (2014) describe the simulation environment *NeuralSyns*, which enables neuroscientists to build, simulate, and visualize large spiking neural networks in a holistic way supporting methods of visual programming. NeuralSyns provides a graphical user interface called *NetBuilder* to support network construction, which in turn generates output that can subsequently be processed by the simulation engine. A driving motivation is to build and parameterize complex network structures without manually handcrafting model descriptions. In contrast to our work, they focus not on the integration of visualization into the computational neuroscientists' workflow, who, in our opinion, are not favoring the replacement of scripting models to using a graphical user interface.

Schmitt and Eipert (2012) presents neuroVIISAS, a generic platform for the integration of data modalities required for the analysis and simulation of biologically realistic neural systems. Furthermore, it allows for the generation of network descriptions tailored to the NEST simulation engine (cf. Gewaltig and Diesmann, 2007). In addition, it provides data analysis capabilities that assist researchers in the exploration of neural dynamics. However, neuroVIISAS does not focus on a tight integration of its use into existing workflows. In contrast to this work, it does not provide a concept for visualization of data while simulations are performed nor a mechanism when a concrete analysis question raises the need for a specifically tailored visualization which then needs to be integrated with existing tools.

Arsiwalla et al. (2015) present *BrainX*^3^, a large-scale simulation system for brain activity with real-time interaction. It builds upon the *iqr* neural simulator and allows for the real-time analysis of network dynamics while simulation is performed. Moreover, it enables users to influence the simulation by inducing activity to network nodes or disconnecting entire brain regions. Albeit the system is specifically tailored to the presented use case it offers an interfacing mechanism to MATLAB for analysis of simulation data. However, the scope of this paper addresses coupling of individual visualizations in order to fit the researchers need in different workflows.

This paper is structured as follows: Section 2.1 describes our users' objectives in modeling simulations, followed by a workflow description of this process in Section 2.2. Section 2.3 is devoted to the requirement analysis where we derive requirements for an architecture based on our observations of the workflow in the previous section. Next, in Section 2.4 we will present the proposed architecture. Section 3 will present results in form of visualizations that utilize this architecture along a use case and present a discussion. Finally, we end this paper with a short conclusion and an outlook on future work.

## 2. Materials and methods

**Figure 1:**
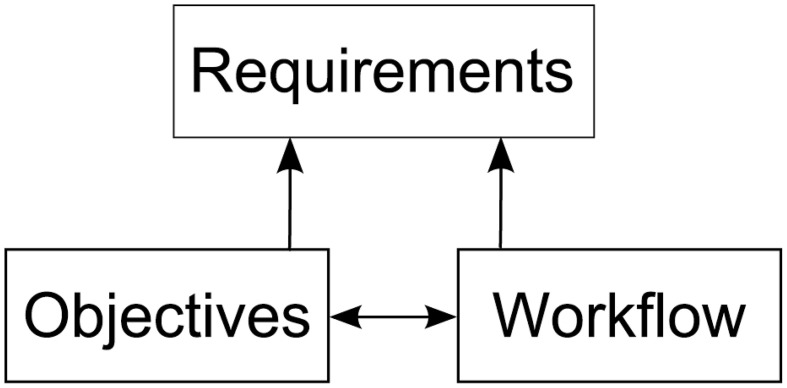
**Depiction of the relationships between research objectives, the workflow and the requirements for a software architecture enabling integration of visualizations into the modeling process**. The research objectives and workflow influence each other. Requirements are derived from both, the objectives as well as the workflow.

In order to derive requirements for a software architecture enabling researchers to integrate visualizations into their workflow of modeling neural simulations, we must first understand how the modeling process is performed. To this end, we asked domain experts to elaborate on their research objectives and workflow steps in the development process of a simulation model. These discussions were oriented along a specific model (the macaque visual cortex), but the described objectives and the workflow have been formulated by the experts in a much broader manner. Thus, we believe these objectives share wider applicability in modeling spiking neural networks. While the research objectives have significant impact on the analysis workflow, the analysis itself can have an impact on the modeling process as well. Therefore, requirements for the aimed architecture supporting these workflows are influenced by both, as depicted in Figure 1.

### 2.1. Research objectives

A simulation of spiking neural networks is based on a mathematical model which forms a basis for studying effects of its behavior, e.g., its dynamics, structure, and size. Modeling is always performed with certain objectives in mind. In order to provide a more concrete example of the modeling process, we oriented these objectives along a neural network for the macaque visual cortex (cf. Schmidt et al., 2014) simulated in NEST.Five objectives were identified:

**Formulate a consistent model definition (O1)**—Derive a consistent definition of a model based on anatomical and electro-physiological data. This data is gathered from publications and databases in order to achieve simulation results in accordance to biological findings, e.g., Stephan et al. (2001), Binzegger et al. (2004), and Markov et al. (2011) to only name a few.

**Systematical parameter study (O2)**—Systematically study the impact of parameters on the dynamics of the model and modify the connectivity within reasonable bounds to reach a stable ground state, close to biological findings, which constitute the verification baseline (cf. Schuecker et al., 2015).

**Investigate emergent behavior (O3)**—Investigate mechanisms underlying differences in firing rates across populations and oscillations emerging through interactions between areas.

**Integrating scales (O4)**—Bridge the gap between large-scale models where each area is represented by a simple dynamical system and detailed spiking models of local cortical networks.

**Research scaling behavior (O5)**—Study effects of scaling the amount of neurons in the model up to realistic sizes of biological systems.

To ensure a systematical approach to investigate these objectives, a workflow is established to incrementally improve the model. The next section will outline these principle steps in more detail.

### 2.2. Workflow analysis

**Figure 2:**
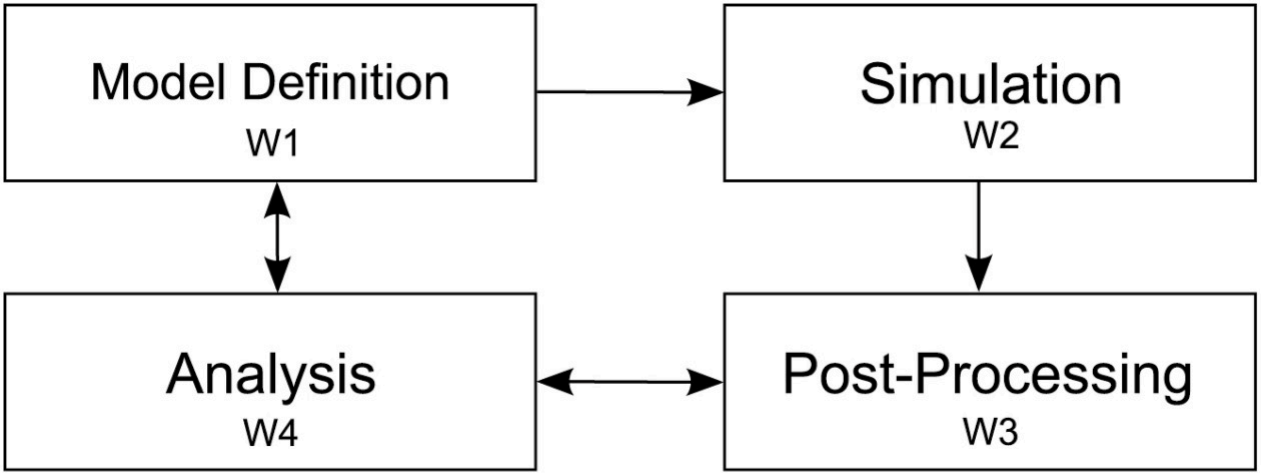
**Depiction of the four elementary workflow steps**. Arrows indicate influences on a particular workflow step to another one. After the model definition (W1) is concluded simulation (W2) is performed. Next, simulation artifacts are post-processed (W3) and passed to the analysis step (W4). Analysis influences the post-processing step whenever, e.g., a new statistical measure is needed to assess system behavior. Analysis also effects the model definition, e.g., to converge the model to biological findings.

To understand the modeling process of researchers in investigating the outlined objectives, we observed four elementary steps while modeling a neural system. Since our collaborators use NEST, we restrict the scope of this description to the workflow resulting from this choice. These elementary steps consist of: first defining the simulation model; second, the execution of the simulation; third, post-processing output resulting from the simulation, and finally exploratory analysis of resulting data artifacts (cf. Figure 2). These elementary steps directly relate to the aforementioned research objectives. The following paragraph will outline these steps:

**Definition of a model (W1)**—A definition of a neural system is encoded in a NEST script and possibly referencing additional resources. This description is either written in SLI, the native simulation language interpreter, or PyNEST (cf. Eppler et al., 2009; Zaytsev and Morrison, 2014), or PyNN (cf. Davison et al., 2009). For parameter studies, multiple model definitions encoding the parameter details are used and subsequently simulated in order to detect change of dynamics in the model (**O2**). Revising model parameters include, e.g., defining synaptic strengths, the relative strength of inhibitory compared to excitatory synapses, the connectivity of neurons, and stimuli to a subset of neurons to induce activity to the network. This task is mainly limited by the turnaround time of a simulation run. Additionally, recording devices are attached to the network model in order to record data from the simulation. These devices can be, e.g., spike detectors, which monitor sets of neurons for spiking activity, or voltmeters, recording voltage traces of neurons. The output of attached recording devices form the raw data for investigating the mechanism governing the behavior of the system, e.g., the firing of populations or oscillations in activity between these (**O3**).

**Simulation of the model (W2)**—When a first model definition has been completed, it is, depending on the computational complexity of the neural system, either simulated directly on the researcher's computer or submitted to a high performance computing machine. Reproducibility is ensured by storing the simulation model as well as all its resources in Sumatra (cf. Davison, 2012). Subsequently, the simulation run is performed.

**Post-process simulation output (W3)**—Once simulation is completed, aggregation of recorded data begins. Due to the distributed nature of NEST computing the model, simulation output is usually scattered over multiple files. This necessitates merging of simulation output as a first post-processing step in order to derive statistical measures quantifying network behavior. These measures will later on be used to conduct parameter studies (**O2**) with the overall aim to converge to a consistent model definition (**O1**). However, the produced post-processing data artifacts are highly dependent on the specific simulation model and the current research questions under investigation. In our example, we focus on a multi-area model where our collaborators also include connectivity information of the network on multiple scales. The model consists of areas, where each area is composed of populations, which on their part are formed of a set of distinct neurons. To calculate statistical measures over populations and areas, this mapping information is required in order to assign neurons to populations and areas. These measures then provide means to investigate the underlying dynamics of populations and areas (**O3**). For instance, statistical measures of interest are activity between areas in unit time, *mean firing rate* of populations and areas, or *correlations of spiking neurons*. In addition to derived data, storing raw simulation output, e.g., spike trains of individual neurons, provide further data artifacts that can be used for visual data exploration.

**Analysis (W4)**—Last, the final step in the workflow applies analysis to the recorded spiking activity, derived statistical measures, and topology information in order to analyze the model's behavior (**O1, O4, O5**). Our proposed architecture is primarily targeting at supporting the analysis process, in particular **O2** and **O3**. Data analysis in this context is a highly volatile process and thereby cannot strictly be mapped to specific instructions that have to be performed in a particular order. This significantly affects a software architecture insofar that it has to be highly flexible in regard to changes in this analysis workflow phase. In fact, due to the sheer unlimited number of combinations of analysis questions, a “one-tool-fits-them-all” approach is most likely a futile development effort. Therefore, a tool ecosystem allowing analysts to link specific visualizations together according to the workflow needs, thus effectively applying the CMV paradigm, is one of the central ideas proposed in this paper.

The described workflow is of iterative nature but not strictly bound to follow each presented step in the same order. Finally, following these observations we conduct a requirement analysis, which we will discuss in detail in the next section.

### 2.3. Requirement analysis

In order to augment the previously described workflow by a software architecture, we derive its requirements essentially from two pillars. On the one hand, we look into the analysis workflow and research objectives with a focus on data modalities resulting from simulations in **W2**, derived measures from **W3**, and existing methods of exploratory data analysis from step **W4**. On the other hand, visualizations, as data consumers, impose certain requirements, e.g., access patterns on data structures, due to their interactivity and real-time rendering capabilities. Based on these, we deduce on requirements for an integration framework. Integration in this context means to bring together post-processing (**W3**) and analysis as part of one workflow (**W4**). In order to support visual analysis, simulation output and its derived statistical measures need to be accessible to visualization applications. In addition, exploratory visual analysis and its specific objectives under investigation often demand specific tools that focus on the particular data sets and scientific question to extract relevant insight. This leads to custom-tailored visualizations that should be embedded in the workflow and can operate as a tool on their own. Nonetheless, scientific questions are not investigated in isolation. Therefore, researchers should be able to link each of these tools together, forming tool chains, in order to meet the current analysis workflow. Thus, an integration framework must make it possible first and foremost, to access, inject, and modify simulation artifacts resulting from **W1**, **W2**, and **W3**. In addition, this data management functionality should directly be integrated into the modeling environment for ease of use and user acceptance, bearing little to no overhead to the modeling effort. On top, it must provide capabilities to link visualizations, which implies clear interface definitions for each visualization component as well as a communication infrastructure to transport data. The integration of the modeling environment aims at a convenient way to setup, configure and drive visualizations within the work environment already used for post-processing data. In the next paragraphs, we will present requirements and a solution capturing the needed functionality.

**Data storage (R1)**—An appropriate storage concept needs to capture simulation output, aggregated from **W2**, model parameters, model-topology, if available, from step **W1** and further statistically derived quantities computed from **W3** in order to support **O1**, **O2**, and **O3**. Ideally, several instances of simulation results can be deposited to allow for, e.g., parameter comparison of the neural systems' behavior or investigate the scaling behavior between downsized neuron models and biologically realistic ones (**O2**, **O4**, and **O5**). Simulation output should be loadable from several sources, i.e., a running simulation, a file stored on disk, or directly injected within the modeling environment. In addition, the storage concept has to be flexible and not tied to a particular simulation model. On top, it has to handle visualization specific data artifacts, e.g., geometry used for rendering, color tables, or configuration settings for view management.

**Data access (R2)**—Data aggregated in a system that meets **R1** needs to be accessed by several consumers synchronously. In particular, mechanisms to access data within the modeling environment has to be provided. Visualizations need means to retrieve data to effectively assist in step **W4**. Because the architecture should allow for visualizations to run on remote systems, data communication over a network needs to be provided. However, since consumers cannot know a priori which data is stored, yet are aware of the data artifacts they process and operate on, an interface to query content from the storage needs to be provided.

**Data modification (R3)**—Modifications on data artifacts resulting from **W1** to **W3** need to be performed at runtime by multiple sources, e.g., a running simulation, the modeling environment, or interactive visualizations. In addition, when changes of data occur, means of notifying consumers operating on this data must be provided in order to ensure distribution of data is consistent. On top, when data artifacts associated with a statistical measure are changed, re-computation of this measure should be triggered.

**Statistical measures (R4)**—Statistical measures of simulation results are one of the main means to analyze model behavior and are the central point of investigation in **W4**. However, these measures and their investigation are highly variable in regards to modification and are added or removed depending on analysis need. Therefore, an architecture ideally includes the computation of statistical measures, with the aim of partially substituting manual triggering computation of these thus assisting in **W3**. Statistical measures have to be recomputed whenever their parameters or implementation changes or the underlying simulation results are updated. In addition, their implementation should ideally be carried out in the modeling environment to allow for rapid prototyping. This leads to more flexibility in adding new measures and empowers researchers to implement these on demand. However, if their evaluation is computationally expensive, the architecture should support exchanging their implementation with more efficient ones.

**Interaction between views (R5)**—Visualizations are tailored to display a subset of heterogeneous data in **W4** to reveal relationships which leads to a multitude of distinct views. On top, there might be interesting relationships depicted in distinct views. In order to reveal these relationships, linking of views is required so that interaction is shared between them. Therefore, synchronization of user interaction is required.

**Controlling views (R6)**—While analyzing simulations (**W4**), views have to be managed. This includes instantiating views, configuring their input and output data, and controlling their individual properties, e.g., to directly jump to a specific point in the simulation, start or stop playback, or reload an updated data set. Moreover, researchers should be able to do so conveniently within the modeling environment.

Based on this requirement acquisition, we introduce an architecture which implements and fulfills the presented requirements in the following section.

### 2.4. Architecture

**Figure 3:**
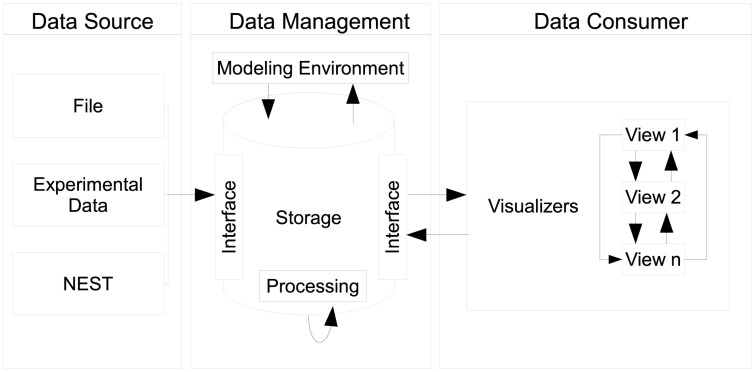
**Depiction of the architecture's design: data sources push raw data to the data management where it is being pre-processed and stored**. The data management acts as single point of access for data consumers and embeds the modeling environment. Multiple consumers, i.e., views can retrieve data from the data management. Consumers share interactions by means of peer-to-peer connections.

Our proposed design centers around three distinct components, as seen in Figure 3, namely data sources, data management, and data consumers. Each component can exist in isolation. However, communication between components explicitly requires the use of an interface which connects individual instances of components together. A special case is the communication of individual views as instances of the data consumer component which directly communicate states between each other.

#### 2.4.1. Data sources and data management

Data sources can be, e.g., the output of recording devices from NEST, experimental data, or a running simulation writing output consecutively per time step. The data management component serves as a data sink for sources to place data into storage in accordance to **R1**. It encapsulates data artifacts from workflow steps **W1**, **W2**, and **W3**. In addition, globally shared data for consumers, e.g., geometry used for rendering, can be deposited. In order to store data a source delivers its content by interfacing the data storage. Consumers query data elements using this interface thus the data management provides a single point of access (**R2**). As a side effect, this enables consumers to act as data transformers processing and subsequently publishing data to the data management.

Modification of content is realized by interfacing the data management (**R3**). Here, only a distinction between whether the modified content should replace a data entity or being appended to the already stored one is necessary. Consider the case where a simulation consecutively ships spike trains per time step to the data management. If the data management can only replace existing spike trains with new ones, this will require all data sources (in this case a NEST simulation) to internally aggregate all previously computed data to ship these consistently, which is unfeasible. By additionally exposing the data management interface to the modeling environment, modelers are able to directly change data in **W4**. Likewise, it eliminates the need to dump simulation output to disk for interfacing visualizations. In order to conveniently populate the data storage, a data source loading simulation output from an HDF5 container (cf. Folk et al., 1999) and forwarding all content has been implemented.

Whenever data is pushed to the data management it can, depending on the data entity received, trigger a processing task. This way, a processing task like the re-computation of a statistical measure can be triggered (**R4**). For instance, whenever spike trains for a neuron population are received computation of the mean firing rate is triggered. Moreover, implementation of measures can be carried out in the modeling environment (**R4**).

In addition to a processing task, notification of change is published to all consumers. Consumers can therefore decide to query for new content or to handle change of data differently. Considering that users can, at any point in time, interact with views it is highly undesirable to reload data thus interrupting analysis. In this case, reloading of data can be postponed until interaction is concluded. Additionally, if a visualization is acting as a data source, data acquisition can be skipped since the change is caused by itself and no reload is required.

#### 2.4.2. Data consumers

Data consumers constitute the last component of this architecture. Consumers are standalone applications, specifically tailored to a particular analysis task. In order to retrieve data consumers connect to the data management component interface (**R2**). Then, requests for data are sent to the data management where they are internally looked up for availability. If available, the data management emits a response containing the data to the requester. Otherwise, an empty respond is forwarded indicating that the request could not be handled. In accordance to **R3**, consumers can change the storage model by acting as a data source. A benefit of decoupling data management from consumers is the preservation of customizations performed in views and the elimination of restarting them. Whenever data is changed, the user's perspective on the data is unchanged, therefore requiring no interaction to reestablish a previous state.

#### 2.4.3. Data synchronization

The proposed architecture distinguishes between two data distribution semantics over a network. The first one is a simple bidirectional communication channel implementing a request-reply pattern (cf. Hohpe and Woolf, 2004) and is solely used for transferring large data chunks between communication partners. The entire data management interface is realized using this distribution semantic. The second distribution semantic implements a *slot concept*, which effectively allows for an event-driven architecture (cf. Michelson, 2006) and is used for light-weight communication, as needed for transferring interaction states between consumers. A *slot* is an asynchronous unidirectional communication channel, which is strongly typed to an event it operates on. A concrete slot is either publishing events or a subscriber to events. Subscribers can be connected to one or multiple publishing slots. Conceptually, connected slots describe a distributed data-flow network (cf. Abram and Treinish, 1995). Slots can be created within the modeling environment or in native application code. In addition, the architecture allows to dynamically reconfigure slot connections at runtime.

**Figure 4:**
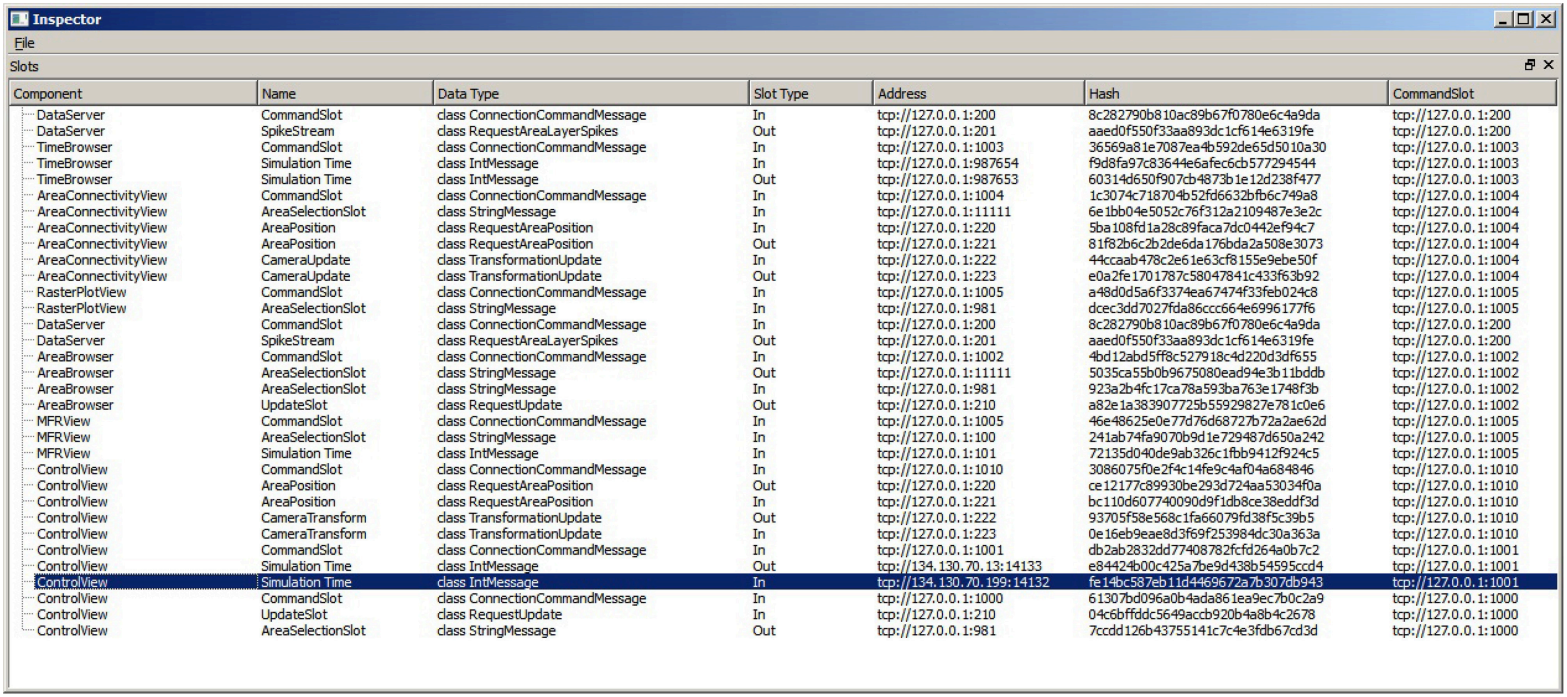
**The ***SlotManager*** is the central registration endpoint for all slots**. It lists all components that registered a slot, shows their name and type and allows for connecting them by double-clicking on their list entry. When a user reconfigures a slot, a control message is emitted that informs the slot to rebind itself to a given address.

Linking user interactions between consumers (**R5**) is realized by directly coupling views by means of a collection of slots. To this end, consumers centrally announce, at their instantiation, functionality they expose via slots. A central slot manager collects all slots currently registered in the system (cf. Figure 4). A linking operation between views is then realized by connecting a subscribing slot of a view to a publishing one of another. Since multiple subscribers can be connected to one publisher, multiple views can be synchronized to, e.g., a selection published by one view. Nonetheless, linking views this way assumes that all consumers share the same understanding (i.e., *semantic*) of how to interpret the content of an event. Currently, the architecture only allows for connecting slots of the same event type, and the implementation of views must adhere *explicitly* to the same interpretation. Explicitly in this context means that a view developer needs to be aware of this restriction and there is no way the system can enforce the same interpretation of an event. There are two possible solutions to enforce this restriction. First, the semantic of an event can be defined to be the type of an event. However, this leads to an explosion of events because even if two events encode the same data structure, they need to be made artificially distinct in order to carry semantics. Second, a more elegant approach, is based on semantic modeling of events. Here, an event is associated with a concept described in an ontology (cf. Wang et al., 2004). Based on this ontology, a semantic reasoner can be used to provide appropriate concepts that can be related. If two concepts are related the system can instantiate additional slots that transform the content of events to the desired one. On top, concept transformations can be encoded as part of the ontology, thus allowing for a flexible integration of views without introducing new events.

Controlling views (**R6**) is realized by exposing slots to the modeling environment in order to connect to views of interest and emit events. Moreover, manual reconfiguration of the data-flow network can be carried out within the modeling environment thus configuring all visualizations conveniently to the current needs in the workflow.

The core architecture is implemented in C++ and is supporting Linux and Windows operating systems. Slot functionality is exposed by providing Python language bindings. This allows for interfacing with any Python based modeling environment and enables development of consumers in either native C++ or Python.

## 3. Results

In this section we present views which utilize the architecture presented in the preceding section. To evaluate the system and to show its applicability, we present it on the basis of the workflow introduced in Section 2.2 with a neural model developed by our collaborators (cf. Schmidt et al., 2014). We assume that workflow step **W1, W2** are concluded and outline the analysis task by introducing four views that operate on the simulation artifacts. Finally, we elaborate on the process of integrating a new view for the analysis tasks into the architecture. Each presented view is a standalone application that is wired via slots in such way to reflect the presented use case. However, they can also be used in different analysis scenarios.

### 3.1. Model development

In our collaborators work, model development is conducted with a combination of SLI for model definition and Python as modeling environment which is primarily used in **W3** and **W4**. To access data for exploratory analysis in **W4** the data management component is instantiated as part of **W3** in the modeling environment. In this scenario, we will focus on introducing the *data explorer view* that enables the inspection of simulation artifacts. Next, we describe the *control view* allowing for the simultaneous exploration of mean firing rates of the simulation model. Following this, we shift focus to the *comparison view* which is linked to user input in the *data explorer view* and assists in the comparison of individual metrics of simulated areas. Consecutively, we present a use case where a simulation script is instrumented in order to visualize spike trains in the *raster plot view* while the simulation is still running.

### 3.2. Data explorer view

**Figure 5:**
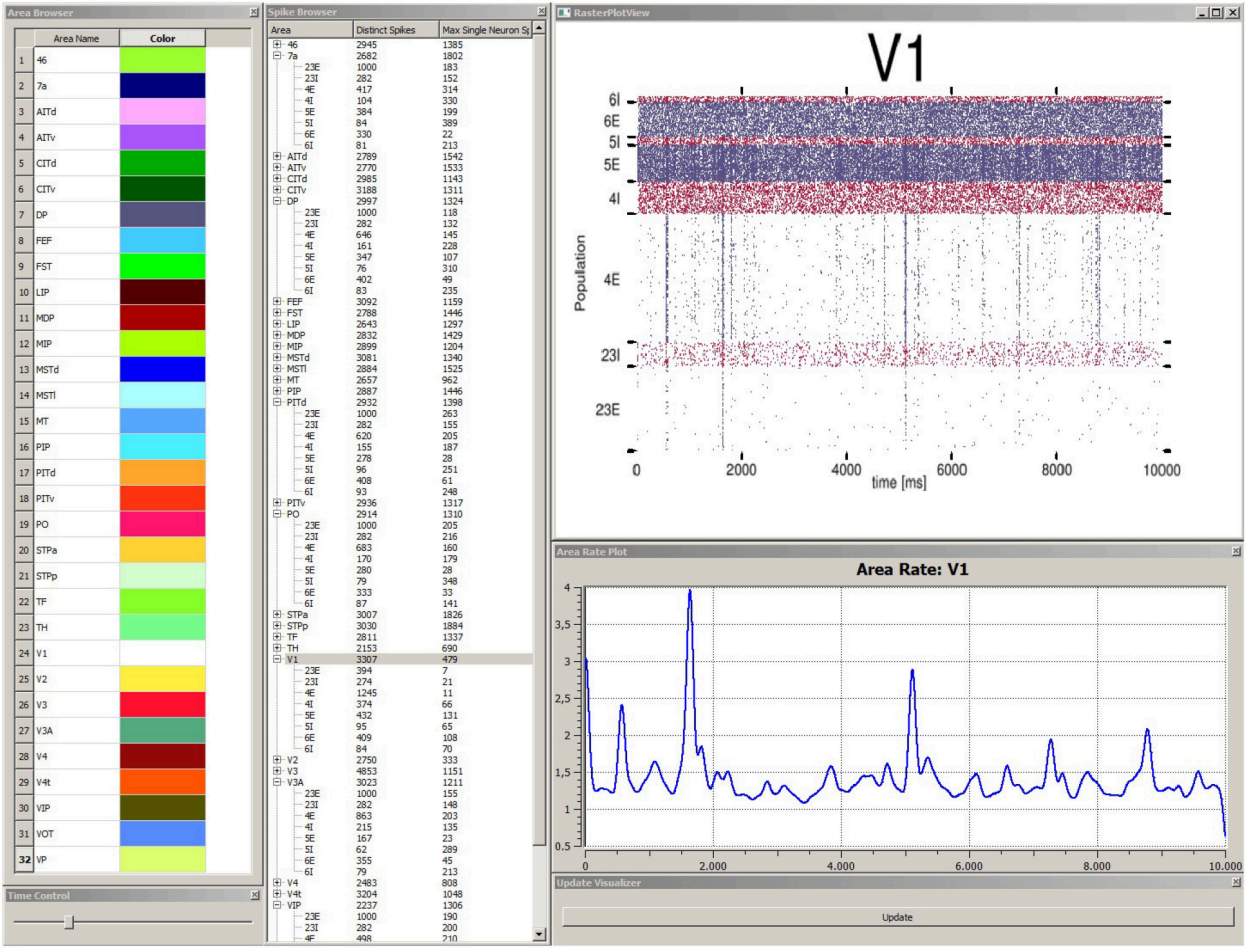
**Depiction of the ***data explorer view*****. This view provides several windows the user can interact with. The area browser (top left) lists all brain areas from the simulation model. A color can be assigned, which is later on used by the control view for area depictions. When the user double-clicks on an area name, the corresponding mean firing rate of this area is displayed as a function plot over time (see area rate plot window) which depicts the difference of spikes per time. The time control window (bottom left) provides a slider to navigate in time, which is also synced with the control view, to allow for convenient navigation in time of the simulation run. The spike browser window lists areas and the name of populations (inhibitory populations are marked in text labels as “I,” excitatory ones “E” respectively) neuron firing statistics. When the user clicks on an area, the raster plot for this area is shown (top right) where red depicts inhibitory populations and blue excitatory ones. The update window (bottom right) allows to manually emit notifications for views to sync data. Data depicted originates from Schmidt et al. (2014).

In order to explore simulation results stored within the data management component the *data explorer view* is started. This allows for a quick overview if data was successfully transferred and measures correctly applied. The *data explorer view* presents the content of the data management as a 2D graphical user interface in several windows (cf. Figure 5). First, it presents a list of all simulated brain areas in the model. Here, colors can be assigned to each area which are used in the *control view*, which will be discussed later, for depictions associated with each brain area. The user can double-click on each area and the mean firing rate as a function plot over time is shown in a separate window. Furthermore, the user can perform multiple selections of areas in this list for comparison in a special view. Second, a spike browser window lists all populations of each area with associated neuron statistics. These include the spike train of the most active neuron and the total number of spikes per population and area. By double-clicking on a population or area, its corresponding raster plot is displayed respectively. Additionally, a time control window presents a slider to conveniently navigate in simulation time which is synchronized with the *control view* in accordance to **R6**. Third, an update window provides a button to manually trigger notifications to views to sync data.

### 3.3. Control view

**Figure 6:**
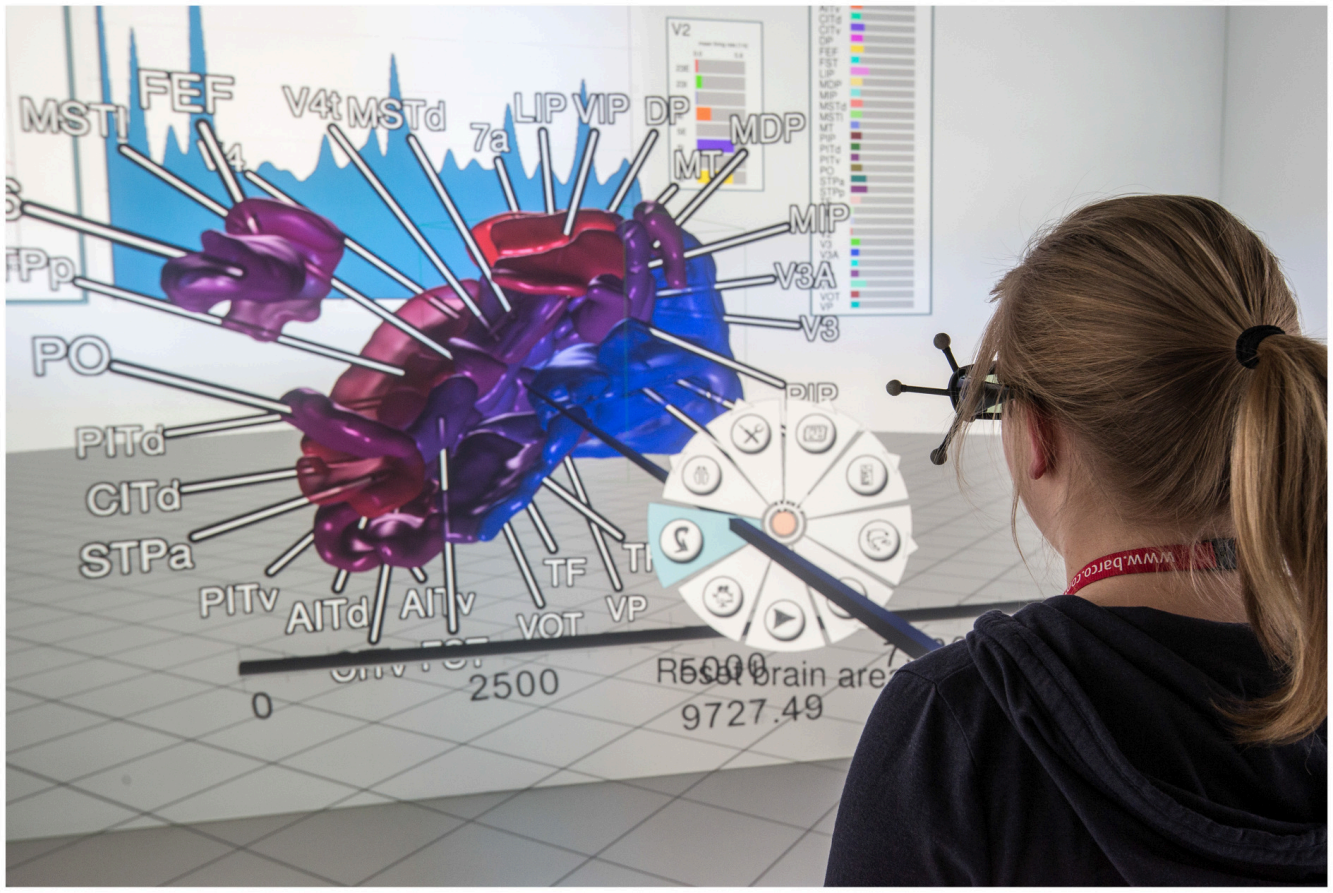
**A user in a CAVE inspects a simulation run using the ***control view*****. Annotations tied to brain areas help to relate brain areas to geometry. Areas are color-coded with regards to their current mean firing rate. 2D panels (background) show statistical measures. System control is realized using extended pie menus (cf. Gebhardt et al., 2013); data depicted originates from Schmidt et al. (2014).

After a quick validation of stored model content, the researcher shifts focus to get an overview of the neural system's behavior over the entire simulation run. Instead of comparing mean firing rates of all areas individually by using the *data explorer view's* plotting window, the *control view* is started. The *control view* provides 3D renderings of all areas using geometry data which relates to the neural model (cf. Figure 6). It provides the advantage of depicting all areas at once and shows their time-varying activity by means of color-coding the geometry of each area. The simulation model of our collaborators studies the macaque visual cortex therefore geometry data of the cortex is used. Since the *control view* is a data consumer it first retrieves geometry data from the data management and queries mean firing rates of areas, populations, the simulation duration and finally spike trains for visualization purposes. Its primary intent is to visualize network behavior to assist in verifying model correctness by making it possible to quickly assess if the simulation run yielded a network in a realistic low-activity state over the entire simulation duration. The *control view* supports different display systems such as a CAVE or a standard 2D desktop system. However, if no such system is available to the researcher, atlas selection of brain areas can be performed in the *data explorer view* by connecting its selection slot as data source to additional consumers. Depending on whether a realistic low-activity state can be observed, further analysis takes place.

### 3.4. Comparison view

**Figure 7:**
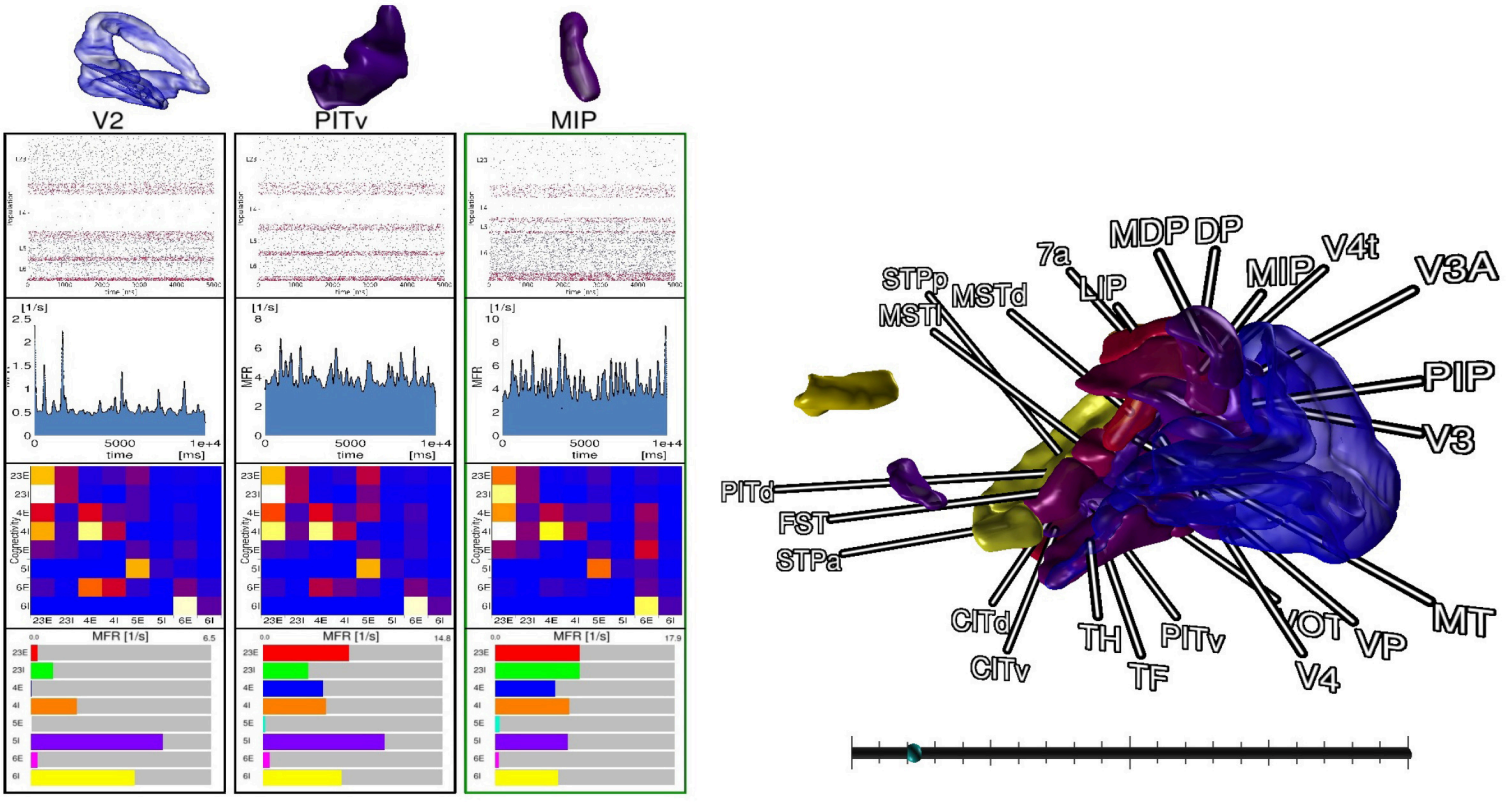
**The ***comparison view*** allows users to compare multiple brain areas in a single 3D view**. Selections performed in the *data explorer view* are synced to a 3D rendering of all areas. By pointing with an input device a selection in 3D on an area is performed, which allows to inspect raster plots of neuron activity, the mean firing rate, the connectivity of populations, and the individual firing rates of its populations next to each other. A slider (bottom right) provides the means to navigate in time. Depicted data originates from Schmidt et al. (2014).

For comparison tasks of neural activity, analysis shifts focus to individual areas and their populations. To this end, the modeler starts the *comparison view*. This view enables researchers to focus on only a subset of areas (cf. Figure 7). The modeler selects areas of interest in the *data explorer* view, but could in principle connect to any consumer exposing selection events via slots, which are then displayed as 3D renderings in the *comparison view*. In addition, panels appear for the selected areas respectively, which depict neuron activity as a raster plot, mean firing rate of individual populations encoded as a moving bar chart, and the connectivity of populations, which is displayed by means of a connectivity matrix. To compare time varying data a slider is provided to navigate in simulation time which is synchronized to the data explorer's time slider. On top, the researcher can point to an area in this view which is then put next to the previous selections in order to conveniently compare them. In this analysis scenario, the modeler is not satisfied with the mean filter used to calculate firing rates. Therefore, the researcher decides to change the convolution kernel within the modeling environment (**R4**) and computes new mean firing rates for all areas. Thereafter, she passes the results to the data management which automatically notifies the *control view* and *comparison view* to retrieve the new data. Since the view on data is preserved, analysis continues without the need to perform the selection task of areas again.

### 3.5. Raster plot view

While analyzing the simulation results, the researcher sees the need to change the model definition. Therefore, a new simulation run is required. However, this time, for a quick hypothesis validation, she is only interested in the spike trains of a particular area in the model and instruments its spike detector. Instead of writing spike trains to disk, she defines a slot which sends all spike events from recording devices of the current time step directly to the data management. Consequently, the spike detector's recordings are redirected to the new slot. While performing simulation, she starts the *interactive raster plot view*, which shows all spike trains currently stored for this area in the data management and updates whenever the simulation produces new data. On top, she is able to pause and continue the solver via slots.

### 3.6. Integrating an LFP view

In the previous paragraph, we have focused on the integration of visualizations into the workflow. However, the presented system offers a variety of additional views which are discussed in more detailed in Nowke et al. (2013). We will now focus on the process to integrate a newly developed view into the system when an analysis question changes. For this reason, we consider a case where local field potential (LFP) measurements for the macaque visual cortex are available from an ife experiment where it is of interest how LFP signals propagate along the cortex surface where measurements were obtained.

**Figure 8:**
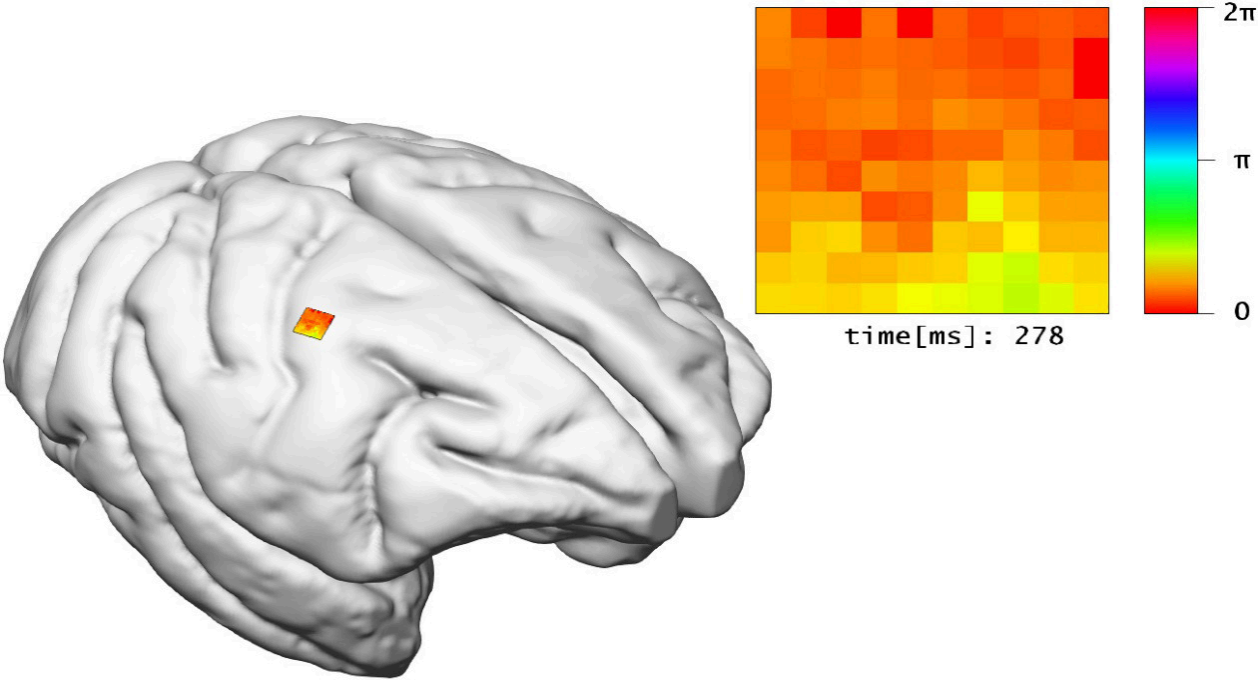
**The ***LFP view*** relates experimental LFP measurements to the location of their recording in 3D space**. In addition, the recorded signals of electrodes are shown in a matrix arrangement, color-coded by their current potential.

The *LFP view* visualizes electro-physiological sum-potentials of neural activity. Signals are recorded extracellular and subsequently low-pass filtered to exclude the direct recording of single action potentials. A 10 × 10 array of micro-electrodes registering LFP signals of the visual cortex while a monkey performs a visual task is used (cf. Ito et al., 2013). One objective of this study is the investigation of correlation between eye-movement and LFP signals. Therefore, this view offers a 10 × 10 color-encoded matrix (cf. Figure 8). Each square represents the filtered output of an individual electrode where color encodes the phase of the LFP signal in the interval [0, 2π] according to a color lookup table. LFP signals are time-varying data, thus the matrix displays changes in color over time. To assure perceptibility of changes in the signal the recording speed is reduced by orders of magnitudes. The 2D matrix is mapped to the 3D surface of the brain and relates to the array's position from the *in vivo* recordings.

In order to integrate the *LFP view* into the architecture, first the data management must be extended to include LFP signal data. Second, a new slot event containing the LFP signal data has to be provided and serialization of its data has to be implemented. Nonetheless, the brain geometry is reused from the *control view* and therefore can directly be queried from the data management. Centralization of LFP data provides a benefit whenever new measurements are made available. Although necessitating the adaption of the data management. In the future, simulations will compute LFP signals. In this setup, the *LFP view* provides a probe to interactively select a region of interest to be visualized comparable to *in vivo* experiments. When comparing the experimental data to simulation results, it might be useful to synchronize the camera position and orientation to, e.g., the control view. For this purpose, two new slots are added to the visualization *LFP view*. The first slot will subscribe to camera transformation updates in order to synchronize the view. The second slot will publish changes of the camera whenever the user interacts within the visualization. Both slots will then be connected.

## 4. Discussion

The focus of the presented architecture is 2-fold: on the one hand is the need to rapidly add new views for exploratory data analysis while on the other hand, a closer integration of visual data analysis into the workflow of modeling neural systems is of concern. The proposed approach embeds visualizations directly into the workflow which has been demonstrated along a scenario of use cases. One of its benefits is the direct control over simulation artifacts within the modeling environment. This simplifies assembling simulation artifacts for visualization purposes (**W3**) and avoids tedious interruptions in the analysis workflow. Additionally, it enables development and integration of new visualizations into rapidly changing analysis workflows by exposing functionality via slots. This mechanism allows visualizations to form tool chains depending on the analysis needs and reconfiguring these at runtime. The key benefit of this architecture is its extensibility and interoperability between consumers that can focus on performing a particular analysis task and be reused by other researchers or consumers in different workflow scenarios. Additionally, it enables data sharing between consumers and offers a mechanism to automatically synchronize changes in data to consumers thus preserving customizations of views, e.g., the user's perspective on the scene. In comparison to the workflow without the presented method, researchers previously had first to assemble visualization artifacts and dump these to disk in a custom data format. Following this, the particular visualization had to be started and configured, e.g., navigating within the data set. Whenever data changed this process had to be redone. Moreover, individual visualizations had no means to communicate, e.g., selection states, therefore requiring these operations to be performed repeatedly for all visualizations to relate data artifacts. Without exposing mechanisms to control views within the modeling environment it was previously impossible to script common analysis tasks, e.g., navigating to a specific simulation time step to study parameter influences. Table 1 presents previously introduced methods and provides a comparison over selected criteria to our method.

**Table 1:** **Comparison of related methods with respect to capabilities: a green checkmark indicates whether a method supports a listed capability and a red cross indicates its absent**.

	**Remote visualization**	**Extensibility**	**Non-relational data**	**Workflow integration**	**3D Visualization**	**Streaming capabilities**
Improvise	×	✓	×	×	×	×
SnapTogether	×	✓	×	×	×	×
CViews	×	×	-	×	×	×
NeuralSyns	-	×	✓	✓	✓	×
NeuroVIISAS	-	×	✓	×	✓	×
BrainX3	-	×	✓	✓	✓	✓
Our	✓	✓	✓	✓	✓	✓

Entries with a blue dash could not be determined from literature. Categories include remote visualization which we define as the possibility to run a view in a network setup. Extensibility we define as the possibility to add views without changing the source code. Non-relational data refers to the capability of handling heterogeneous data. Workflow integration means to control consumers within the modeling environment. 3D visualization allows for 3D rendering of spatial data and streaming capabilities indicate whether the method is known to stream data from solvers.

We presented a use case where linking user selections to different views is required (**R5**) as presented in Sections 3.2 and 3.4. While a concept to systematically formalize user interaction and its transformations to views is difficult to realize, the architecture forms a basis for advanced semantic linking concepts in the future. One key step toward a realization is a semantic description of simulation artifacts. Next, operations and user intents on these need to be formalized. Based on this description, the system can present a choice of suitable candidates of views which operate on a simulation artifact and fulfill the user's analysis intent. Moreover, by exposing functionality of consumers via slots combined with semantic descriptions thereof analysis intents can be matched against these to extract a collection of consumers that chained together reflect the desired workflow. In addition, based on the semantic description of functionality and required interaction to operate views, transformations can be inferred. For instance, given a user selection of a spike train in a raster plot view propagated via a slot to a consumer operating on populations and there exists a relation between the concept of a spike train to the concept of a population, the system could automatically instantiate a transformer. The transformer consists of two slots where the first accepts a spike train and maps its input via a transformation encoded as part of the concept to its second slot. Then, the system can automatically reconfigure the data-flow to interconnect the transformer between the raster plot view and the consumer accepting a selection of populations. This approach allows for flexible scaling of simulation artifacts and interactions whenever new consumers are added. Thus, the presented architecture and its associated visualizations represent a potential solution for a coordinated multiple views framework assisting visual analysis tasks while modeling neural networks in computational neuroscience.

A current drawback of the architecture's implementation is the need to copy stored data entities to send them over a network regardless of consumers running on the same machine. However, using techniques like interprocess communication and shared memory tables, optimization strategies for this case are possible to implement as future work. The communication mechanisms provided by slots is currently explicit; meaning that the presented system cannot restrict users to connect, by mistake, slots which do not share the same interpretation of events. Nevertheless, the system allows for the development, integration and adaption of visualizations to the rapidly changing analysis workflows and can be improved to steer clear of its current limitations. A major benefit of exposing functionality by slots and providing language bindings to the modeling environment is an API allowing researchers to integrate their own analysis tools in Python or C++ and reusing already integrated ones. A video demonstrating the architecture is included as part of the supplementary material.

## 5. Conclusion and future work

In this paper we have presented overall objectives for modeling neural system simulations and inspected a resulting workflow. From this starting point we have inferred requirements for an architecture supporting the analysis process with interactive visualization techniques and have presented an architecture covering these requirements. In addition, we have demonstrated its applicability along a use case and showed where interactive visualizations can assist in modeling neural simulations. Moreover, we have described the integration of a new visualization into the system when analysis shifts focus. Finally, we have presented a discussion elaborating on the benefits of our approach and hinted at further development with regard to semantic linking of views.

As future work we would like to couple NEST more tightly to this integration approach by implementing recording device proxies which can directly act as data sources for the data management component. This would allow for the inspection of network behavior while the simulation is still computed. To readily investigate the impact of parameter changes, we would like to develop visualization methods focusing on comparing two or more simulation runs by, e.g., computing difference signals, highlighting changes in connectivity within the model, and assist in the evaluation of firing behavior of populations and areas. Moreover, we are interested in exploring further use cases evaluating the architectures applicability and present visualizations more thoroughly tied to a concrete neuroscientific workflow. Finally, we would like to develop methods to semantically describe the input and interaction techniques of views in order to allow for a flexible integration and user controlled coupling between them. This would allow for a simple integration and reuse of views depending on the rapidly changing workflow.

## Author contributions

CN: Conceived and implemented the described architecture and has written all major parts of the article. DZ: Contributed the LFP-View and has written the paragraph about it in the paper. BW, BH, and DZ: Substantial revised the article, contributed to the ideas presented in the article and supported the definition of the scientific methods. TK: Revised the article and presented valuable input to the manuscript. AP: contributed to the underlying spike streaming concept for NEST simulations.

### Conflict of interest statement

The authors declare that the research was conducted in the absence of any commercial or financial relationships that could be construed as a potential conflict of interest.
